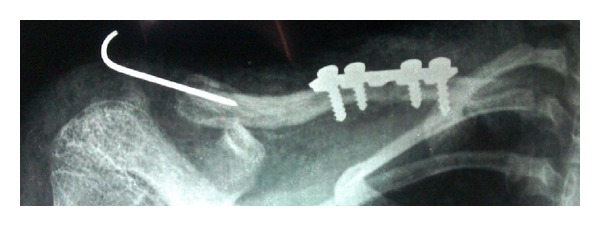# A Rare Case of Segmental Clavicle Fracture in an Adolescent

**DOI:** 10.1155/2013/248159

**Published:** 2013-02-10

**Authors:** Arup Kumar Daolagupu, Parag Jyoti Gogoi, Srikanth Mudiganty

**Affiliations:** Department of Orthopaedics, Silchar Medical College & Hospital, Silchar, Assam 788014, India

## Abstract

Clavicular fractures commonly occur in adults and children. The usual mechanism of injury is a fall on the outstretched hand or direct trauma. The usual site of these fractures is the mid clavicle with lateral end and medial end clavicular fractures being less common, respectively. Segmental clavicular fractures have been reported in the literature; they usually occur at the medial and lateral ends and tend to occur in adults. Bipolar clavicular injuries involving medial and lateral ends have also been reported rarely but all in adults. We report a very rare case of segmental clavicular fracture involving the mid clavicle and lateral end in an adolescent caused by direct trauma. The management of segmental clavicle fractures has not been clearly outlined although operative intervention is indicated. We report the successful management of segmental fracture clavicle in an adolescent and discuss the relevant literature.

## 1. Introduction

Clavicle is an S-shaped subcutaneous bone which increases the band ring power of the shoulder. Clavicular fractures account for approximately 2% to 5% of all fractures in adults and 10% to 15% in children [1]. The incidence of clavicle fracture in adolescents and adults has been reported to be 29 to 64 per 100,000 persons annually [2]. Midshaft clavicular fractures are the most common, ranging from 69% and 82%, distal fractures comprise 21% to 28%, and proximal fractures occur between 2% and 3% [3]. Clavicle fractures exhibit a bimodal age distribution occurring in young males due to direct trauma and in elderly patients due to domestic falls.

Fractures of clavicle are sustained as a result of direct trauma or fall on the outstretched hand. Although fall on the outstretched hand is thought to be the most common mechanism of injury, Stanley et al. in their analysis of 122 clavicle fractures found in 94% of cases the mechanism of injury to be direct trauma [4]. Mid clavicle fractures may also occur due to direct force over the shoulder forcing the clavicle over the first rib.

Segmental clavicular fractures have been reported in the literature involving mid shaft and lateral end [5, 6]. There are isolated case reports of bipolar clavicle fractures (combined medial and lateral end clavicle fractures), all occurring in adults [7–11]. We report a case of segmental clavicle fracture in an adolescent caused by direct trauma. The management of segmental clavicle injuries is discussed.

## 2. Case Presentation

A 12-year-old boy presented to the emergency department with pain over the right clavicle following a history of fall from height. There was no open wound. Physical examination revealed tenderness over the entire clavicle. Movements of the shoulder joint were restricted secondary to pain in the clavicle. There was no evidence of neurovascular deficit and no other significant injuries. 

**Figure 1:**
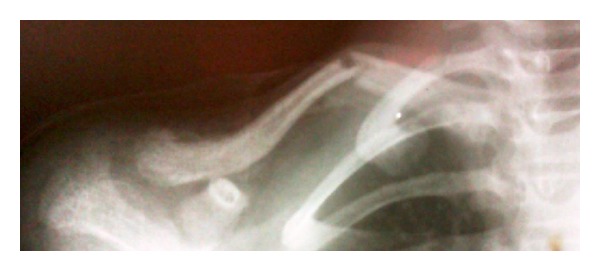


Radiographs showed a segmental fracture clavicle with a mid shaft and a lateral end clavicle fracture. (Figure 1). 

Two days after the incident, surgery was performed under general anaesthesia. Open reduction and internal fixation of mid clavicle fracture with plate fixation was done. Lateral end clavicle fracture was treated with closed reduction and K-wire fixation. (Figure 2). 

K wire was left outside the skin and was removed after 3 weeks. Mobilisation and physical therapy for shoulder movements with active exercises was started. Bony union was achieved by 6 weeks and the patient had pain-free full range of movements of shoulder joint. 

## 3. Discussion

Segmental clavicle fractures are rare. Of the 614 clavicle fractures reviewed by Throckmorton and Kuhn only 0.8% had segmental injuries [12]. Bipolar injuries (combined proximal and distal fractures) are extremely rare with isolated cases being reported, all in adults. 

Clavicle fractures are usually caused by a direct trauma to the shoulder [4] but the mechanism of injury leading to a segmental clavicle fracture is not well understood. It has been postulated that they occur due to two separate concurrent forces [7, 11]. Perhaps our patient sustained two separate successive injuries to his clavicle at the time of fall. Although these fractures have been described in adults, to the best of our knowledge such fractures have not been reported in adolescents. 

Although the majority of clavicle fractures are managed nonoperatively, specific indications exist for operative intervention [13]. Segmental long bone fractures are considered unstable injuries with the risk of nonunion and nonoperative approach being considered unacceptable [7]. The clavicle forms an important part of the shoulder girdle and is vital for the normal functioning of the upper limb. Non-union of the clavicle fracture would result in considerable functional deficit. With meagre literature regarding segmental clavicle injuries, no consensus exists about the management of these fractures with some case reports advocating a non-operative [9, 10] while others an operative approach [7, 8]. 

The optimal treatment needs to be individualised based on the patient and fracture pattern. 

The purpose of this paper is to highlight the rarity of this case and the fact that segmental fracture of clavicle should be kept in mind whenever a clavicular fracture is diagnosed in adults as well as in adolescents. The treatment should be based on the individual fracture pattern and patient characteristics.

## Figures and Tables

**Figure 2:**